# Modeling Verdict Outcomes Using Social Network Measures: The *Watergate* and *Caviar Network* Cases

**DOI:** 10.1371/journal.pone.0147248

**Published:** 2016-01-29

**Authors:** Víctor Hugo Masías, Mauricio Valle, Carlo Morselli, Fernando Crespo, Augusto Vargas, Sigifredo Laengle

**Affiliations:** 1 Department of Management Control and Information Systems, Universidad de Chile, Santiago, Chile; 2 Centre international de criminologie comparée (CICC), École de criminologie, Université de Montréal, Montréal (Québec), Canada; 3 Faculty of Economics and Business, Universidad Finis Terrae, Santiago, Chile; 4 CEDYTEC, Dirección de Investigación, Universidad Bernardo OHiggins, Santiago, Chile; 5 Departamento de Diseño y Manufactura (DIMA), Universidad Técnica Federico Santa María, Viña del Mar, Chile; Universidad de Alicante, ITALY

## Abstract

Modelling criminal trial verdict outcomes using social network measures is an emerging research area in quantitative criminology. Few studies have yet analyzed which of these measures are the most important for verdict modelling or which data classification techniques perform best for this application. To compare the performance of different techniques in classifying members of a criminal network, this article applies three different machine learning classifiers–Logistic Regression, Naïve Bayes and Random Forest–with a range of social network measures and the necessary databases to model the verdicts in two real–world cases: the U.S. *Watergate Conspiracy* of the 1970’s and the now–defunct Canada–based international drug trafficking ring known as the *Caviar Network*. In both cases it was found that the Random Forest classifier did better than either Logistic Regression or Naïve Bayes, and its superior performance was statistically significant. This being so, Random Forest was used not only for classification but also to assess the importance of the measures. For the *Watergate* case, the most important one proved to be *betweenness centrality* while for the *Caviar Network*, it was the *effective size of the network*. These results are significant because they show that an approach combining machine learning with social network analysis not only can generate accurate classification models but also helps quantify the importance social network variables in modelling verdict outcomes. We conclude our analysis with a discussion and some suggestions for future work in verdict modelling using social network measures.

## Introduction

Although modelling criminal trial verdict outcomes is a classic problem in predictive criminology [1], building verdict classification models for criminal networks is a relatively new area of research. This paper compares the performance of different analytic techniques for addressing the problem of verdict outcome classification using machine learning and social network measures.

The scientific investigation of social networks in criminal organizations is a branch of quantitative criminology that generates knowledge regarding such networks through the analysis of links between network members [2]. Such an analysis requires data that, unlike the information normally employed by criminologists, bears directly on these membership ties. By examining these data, the researcher can explore in detail the social behaviour of criminal groups and organizations [3–12] and terrorists operations [13–15]. This focus on the ties or links between group members is what accounts for the success of social network analysis in the study of criminal organizations [16–25].

The problem of verdict outcome classification in particular is of great interest to various actors in criminal justice systems, and especially to forensic criminologists faced with the task of converting a set of data into evidence of a network’s criminal conduct. To our knowledge, however, only three academic studies have analyzed the relationship between verdicts and social network measures: the pioneering work by Baker and Faulkner [26] and, more recently, the papers by Faulkner and Cheney [27] and Morselli, Masías, Crespo and Laengle [28]. These authors have used different sets of social network measures to test their relationships with verdict outcomes. Their general conclusion is that social networks have much potential for constructing models that can successfully predict verdicts.

Valuable though these three analyses are, they all confine their methodologies to the use of Logistic Regression as a data classifier. Studies in other contexts comparing different classifiers have shown that that their performance can vary significantly depending on the data domain they are applied to [29–35]. This suggests that classification techniques other than Logistic Regression should be evaluated to determine how well they perform comparatively with criminal network data.

The present article is an attempt to carry out just such comparisons. Two real-world cases will be used for the purpose: (1) the *Watergate Conspiracy* (WC), the American political scandal of the 1970’s; and (2) the *Caviar Network* (CN), a now-dismantled international drug trafficking ring that was based in Montréal, Canada. The classifiers whose performance will be evaluated and compared in addition to *Logistic Regression* (LR) [36] are *Naïve Bayes* (NB) [37] and *Random Forest* (RF) [38]. Our contribution consists principally in demonstrating that an approach combining machine learning with social network analysis not only can generate accurate classification models but also helps quantify the importance social network variables in modelling verdict outcomes. Both of these conclusions are new findings in the field of criminology and penology.

The remainder of the article is organized into four sections. Section 2 reviews the relevant literature; Section 3 presents the methodology, the data, the social network measures, the analysis and the models obtained; Section 4 sets out the results separately for the two cases studied and the importance of each network measure; and finally, Section 5 discusses the results and a number of specific issues raised by the analysis and states our final conclusion on the performance of the three classifiers in modelling verdict outcomes.

## Literature Review

As noted in the Introduction, there are three case studies in the literature that investigate verdict classification based on social network measures [26–28]. The specific problem these papers attempted to address is the following: given a set of evidence or data on the relations between individuals in a social network suspected of criminal activity, what can be inferred with a certain degree of confidence regarding their guilt or innocence? Traditionally, the data criminologists work with do not include information on such relations. By contrast, the relatively new social network approach focuses explicitly on these interdependencies.

The three studies explored the predictive power of various measures of centrality, which “quantify an intuitive feeling that in most networks some vertices or edges are more central than others” ([39] [p.16]). All three found the centrality of a criminal network member to be correlated with verdict outcomes. In [26], the earliest of the articles, Baker and Faulkner investigated illegal networks in the *Heavy Electrical Equipment Industry* (HEEI) that were involved in conspiracies to fix the prices of switchgears, transformers and turbines. Their analysis chose 78 individuals from 13 companies who directly participated in the price-fixing, 37 of whom were eventually found guilty or pleaded no contest (*nolo contendere*). The authors discovered that the centrality degree indicator, which measures the number of direct contacts an individual has with others, had a positive and significant relationship to the verdict. As centrality degree increased, the probability that a given agent was found guilty increased as well. Using this metric Baker and Faulkner were able to identify 87% of the individuals who were found guilty and 78% of those who were found innocent.

The second case study [27] analyzed the Watergate scandal [40], a highly complex criminal case in which various individuals were found guilty or innocent and a number of the sentences handed out were subsequently increased, reduced or revoked [41, 42], but initially 7 persons were convicted. The authors showed that the betweenness centrality indicator [43], which measures the number of shortest paths from all vertices to all others that pass through a given network member, contributed significantly to the guilty verdict classifications. As betweenness centrality increased, the probability that a given conspirator was found guilty also increased [27].

The third case study, a collaborative effort by Canadian and Chilean researchers, analyzed the Caviar Network, a former Canada-based drug trafficking operation as noted in the Introduction [28]. This work is particularly revealing because unlike the other two cases, it used data collected from real communications between the network members. The police investigation of the network resulted in the arrest of 25 individuals, of whom 22 were charged and 14 found guilty. The study found that the out-degree centrality indicator [44], which measures the agent out-flow communication edge, made a significant contribution to the verdict classifications. As out-degree centrality increased, so did the probability that a given conspirator was found guilty [28]. The findings of this analysis and the two other studies just discussed are summarized in Table 1, showing the different measures tested in each case and the statistical significance of the results.

**Table 1:** Results of three case studies testing social network measures as predictors of verdict outcomes.

Case Study	HEEI		CN		WC	
Source	[26]		[28]		[27]	
Social Network Measure	Tested	Significant	Tested	Significant	Tested	Significant
*Authority centrality*	No	No	Yes	No	No	No
*Betweenness centrality*	No	No	Yes	No	Yes	**Yes**
*Closeness centrality*	Yes	No	Yes	No	No	No
*Degree centrality*	Yes	**Yes**	Yes	No	No	No
*Hub centrality*	No	No	Yes	No	No	No
*In–Degree centrality*	Yes	No	Yes	No	No	No
*Out–Degree centrality*	Yes	No	Yes	**Yes**	No	No

In brief terms, the three studies found empirical support for the hypothesis that social network indicators show considerable potential for modelling verdict outcomes. This suggests that the degree of responsibility of an individual in a network can be related to their networked behaviour. These are new findings given that most previous studies have attempted to make predictions based on social, demographic, economic or ethnic variables, or on variables relating to the functioning of the judicial system, among other factors [45–52].

The demonstration of this working hypothesis opens up an area of research that raises various predictive analysis problems. One of these problems relates to the data. A promising approach to the data sets is provided by the NB and RF classifiers referred to earlier. Although there appear to be no previous works applying these classification techniques to the issues investigated here, some studies have shown that NB and RF perform better than LR in the identification of terrorist attacks [53–57]. For example, Graham et al. [58] reported that NB correctly identified roughly 80% of the perpetrators of terrorist group events in the Philippines. In another paper comparing the three classifiers, Hill et al. [54] found that RF outperformed LR and NB in correctly identifying the guilty parties in one of the world’s major terrorism hot spots.

With the above considerations regarding the state of the art in mind, the next section describes the experimental method adopted for the present study.

## Materials and Methods

### Methodological Setup

**Fig 1:**
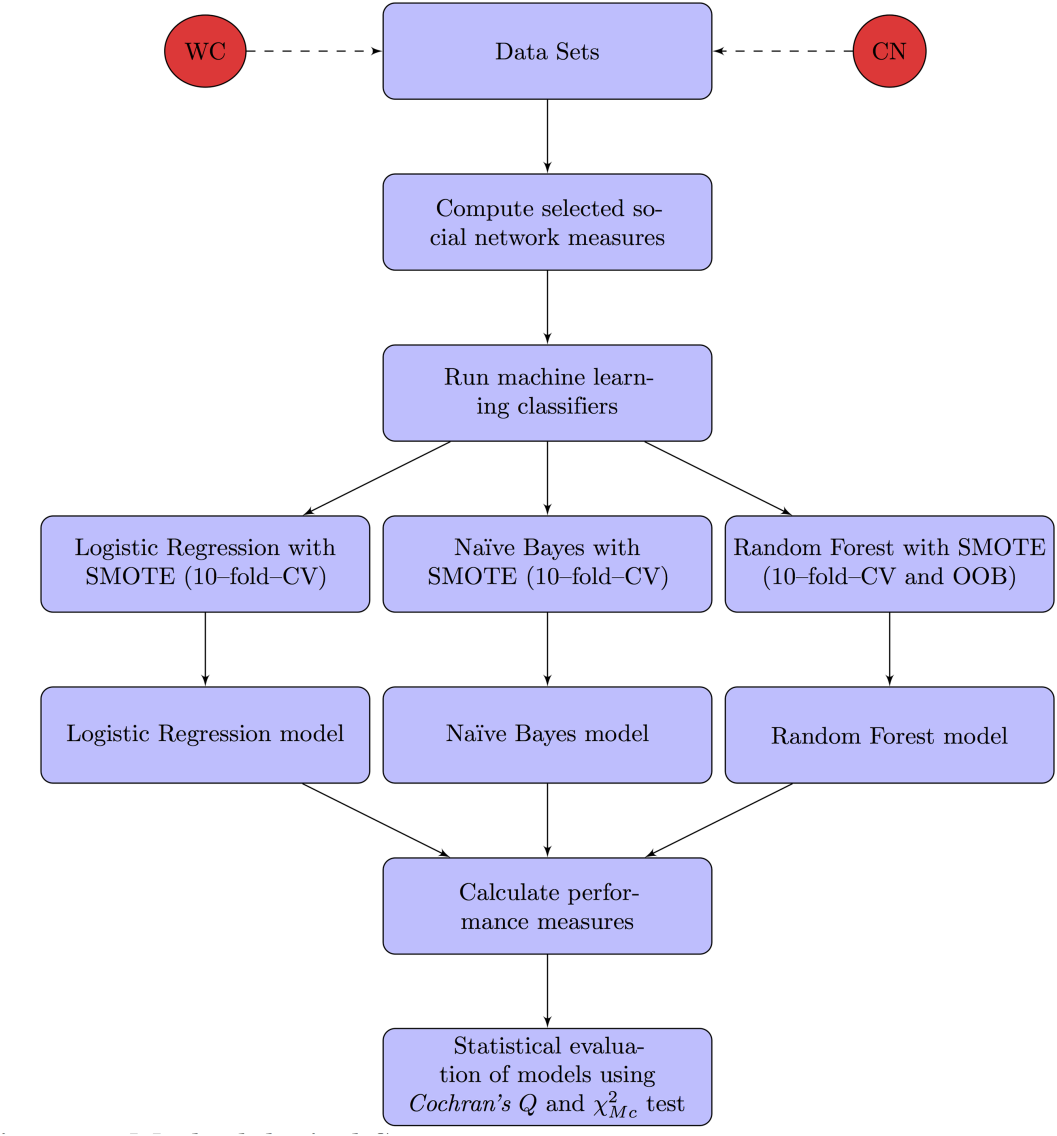
Methodological Setup.

The method and strategy followed in our comparative analysis is depicted in the flowchart in Fig 1. A set of 16 social network measures were chosen to be the independent variables and in both cases the dependent variable was the verdict outcome, which categorized each criminal agent binarily as either guilty or innocent. The next step was to calculate the various social network measures using the original data sets for the WC and CN cases. Predictive models based on LR, NB and RF classifiers were then constructed. To address the class imbalance in the two data sets, the *Synthetic Minority Oversampling Technique* (SMOTE) was used [59]. The models were validated using the *10–fold Cross–Validation* (10–fold–CV) technique [60]. To compare their respective performances, various performance measures were applied. These included *Accuracy*[61], *Precision*[62], *Recall*[62], the Area under the ROC curve (*AUC*) [63, 64] and the *Matthews Correlation Coefficient*[65]. Finally, a series of tests using *Cochran’s Q* and *McNemar’s* test statistic ($\chi_{Mc}^{2}$) [66, 67] were conducted to determine whether the differences recorded were statistically significant. More detailed information on these various steps is given in the following subsections.

### Data sources and networks for analysis

The data sets for the WC and CN cases are described below:

#### WC Working Web

**Fig 2:**
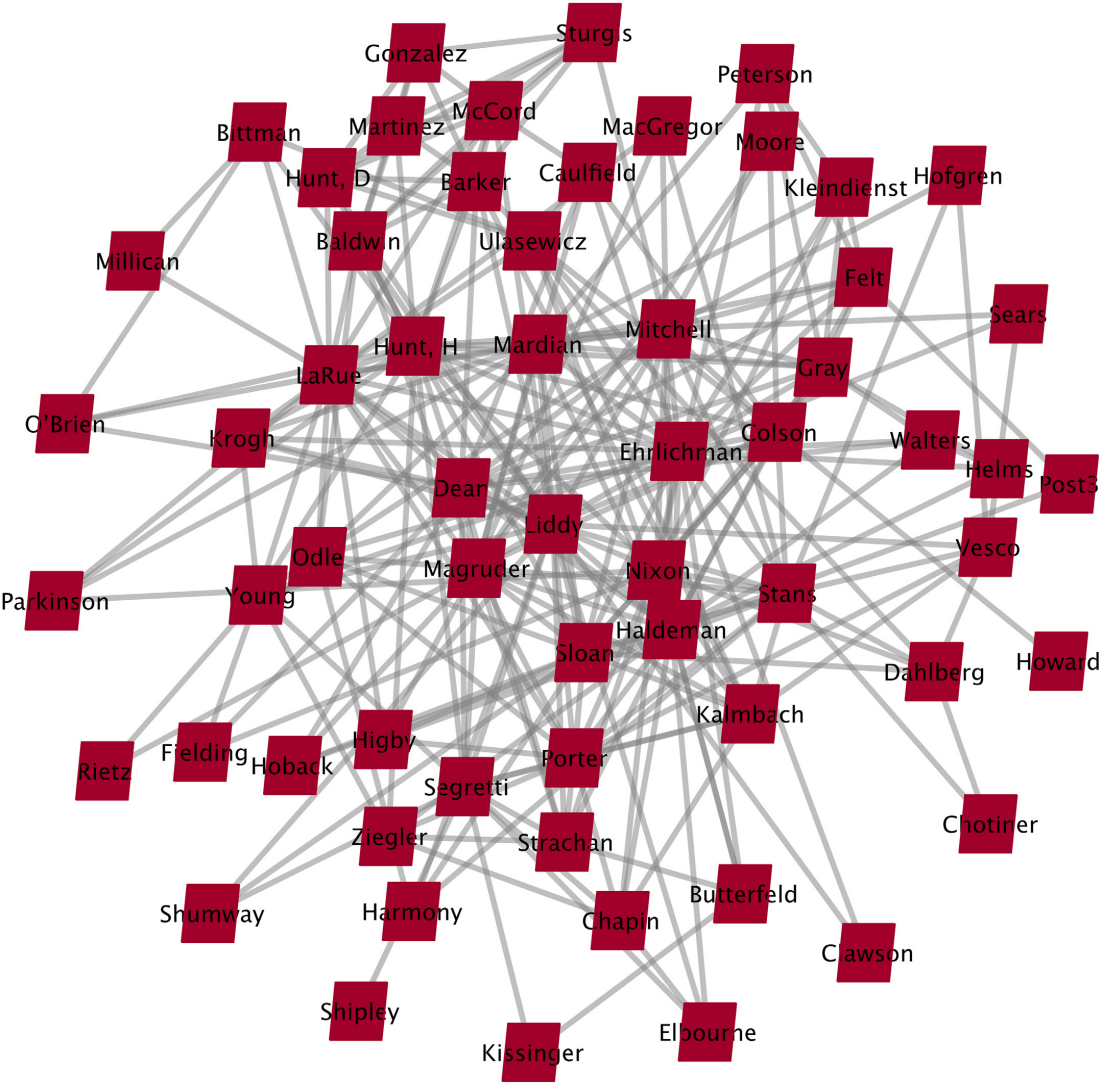
The Working Web of Watergate (*n* = 61). The coding was used to map these relationships, generating what the authors [27, 68] call a “Working Web of Watergate”. If two agents (co-conspirators) worked together on illegal activities (i.e., illegal espionage, money laundering, or sabotage) a 1 was entered in the corresponding cell of an adjacency matrix, otherwise a 0 was entered. Thus, the link values in the completed matrix were binary.

The source of our data for the WC case was the documentary research carried out by Faulkner and Cheney [27, 68]. Each author individually coded the information they collected to establish the relations between network members and determine who did what with whom in the various Watergate activities, and then compared their results. Finding that they agreed 100% of the time on which actors worked with whom, they concluded that their coding was highly reliable. A sociogram of the WC data set is shown here in Fig 2.

#### CN Communication Flow

**Fig 3:**
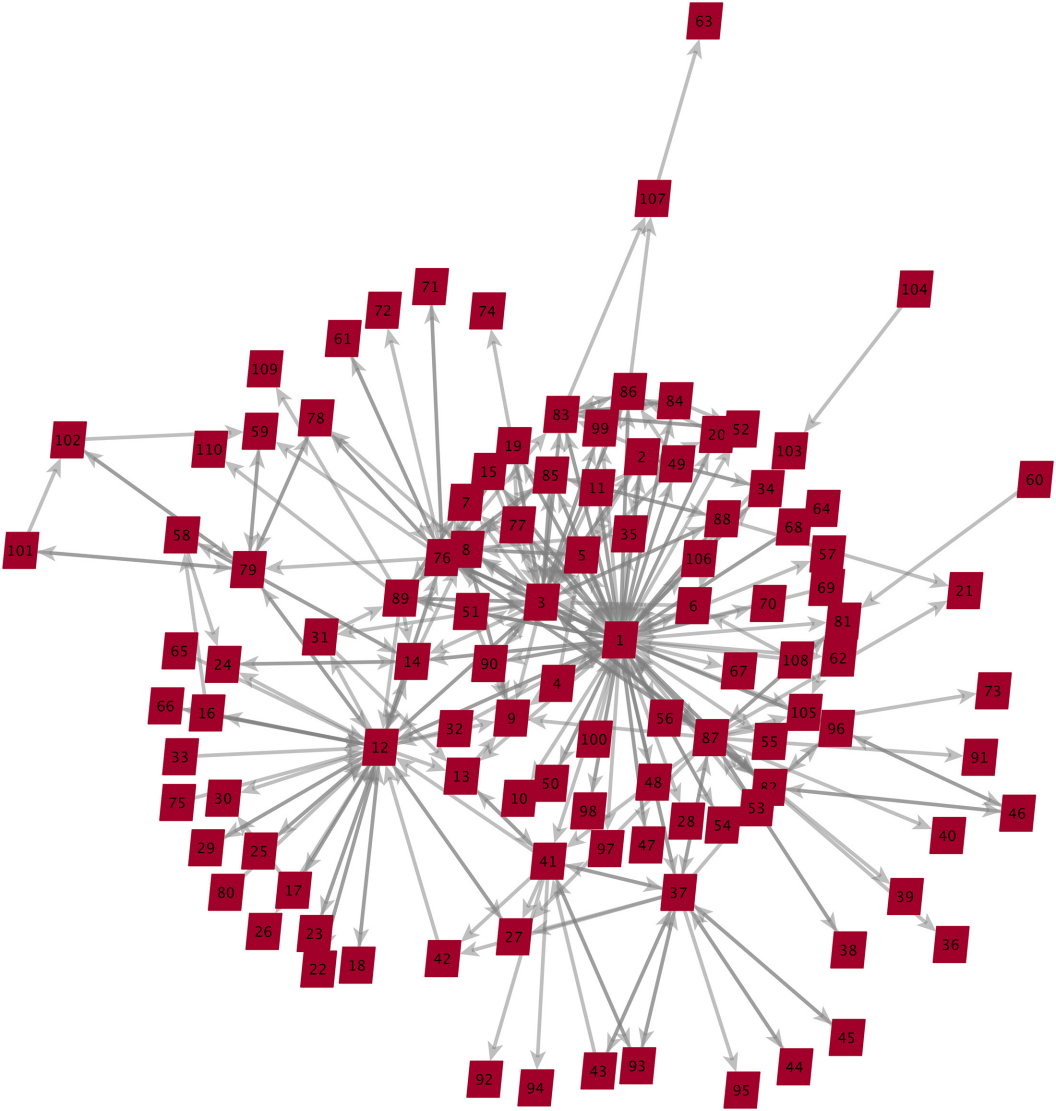
CN communication flow (*n* = 110). The data set maps a communication flow between agents indicating who was communicating with whom. Each cell of the adjacency matrix created for the case contains the number of times the corresponding pair of agents communicated with each other. So as not to reveal the identity of the monitored individuals, an identification number was assigned to each. Thus, the link values of the completed matrix were non–negative integers.

The source of our data for the CN case was the documentary research conducted by Morselli [69], who collected evidence derived from electronic surveillance transcripts presented in court during the trials of some of the network participants. The more than 1,000 pages of transcripts released to the public revealed the communication flow that existed inside the network of drug trafficking operations. A sociogram of the CN data set is shown here in Fig 3.

### Compute selected social network measures

The 16 social network measures serving as the independent variables are briefly described in Table 2. Some of them were found to be statistically significant in the previous studies (see Table 1) while others were tested here for the first time. Also, whereas all 16 measures could be calculated for CN, only 12 could be for WC because of the binary or non–binary nature of the data or the symmetry or asymmetry of the matrix (S1 Supporting Information).

**Table 2:** Definition of independent variables.

Measure	Meaning [70]	Reference
***Degree centrality***	The number of neighbours of a agent	[44]
***In–Degree centrality***	Agent in–flow communication edge	[44]
***Out–Degree centrality***	Agent out–flow communication edge	[44]
***Eigenvector centrality***	Degree of connected agents who are themselves connected to many players	[71]
***Authority centrality***	An agent is authority–central to the extent that its in–links are from agents that have many out–links	[72]
***Hub centrality***	A node is hub–central to the extent that its out–links are to agents that have many in–links	[72]
***Betweenness centrality***	Across all agent pairs that have the shortest path containing the player, the percentage that pass through the player	[43]
***Information centrality***	Calculates the Stephenson and Zelen information centrality measure for each agent	[73]
***Triad count***	Number of triads centred at the agent	[74]
***Interlockers***	Interlocker are agents that have a triad count (the number of triads each node is in) that is greater than the mean plus one standard deviation	[74]
***Radials***	Radial are agents that have a triad count (the number of triads each node is in) that is less than the mean minus one standard deviation	[74]
***Clique count***	The number of distinct cliques to which each agent belongs	[75]
***Constraint***	The degree to which an agent is constrained by its current communication network	[76]
***Effective network size***	The effective size of a agent’s communication network based on redundancy or ties	[76]
***Clustering coefficient (local)***	Density of the agent’s ego network, which is subgraph induced by its immediate neighbours	[77]
***Simmelian ties***	Number of ties with strongly, reciprocally connected players when there are one or more third–party agents who commonly have strong and reciprocal edges to themselves and the connected agent	[78]

### Run machine-learning classifiers

In what follows we describe some of the techniques used in building the models, balancing the classes, and running, validating and evaluating the models.

#### Classifiers

The three machine-learning classifiers used in this study described below:

- The LR classifier learns probability functions of the form *P*(*Y*|*X*), where *Y* is the class variable and *X* the attribute vector [36]. LR assumes a parametric function for the distribution of *P*(*Y*|*X*), and based on the training data it estimates the distribution’s parameters. The distribution is usually a logistic function, thus justifying its name as well as ensuring the probabilities range between 0 and 1. *P*(*Y*|*X*) can then be a linear combination of the predictor attribute vector.
- NB computes the conditional *a posteriori* probabilities of a class variable given some set of predictor variables using the Bayes rule [37]. It is simple to implement and has proven to perform very well with a variety of data types in supervised learning settings even though it implicitly assumes independence of attributes [79]. NB theoretically works best when there are independent features as predictor variables. However, as has been pointed by Rish, “The naive Bayes classifier greatly simplify [sic] learning by assuming that features are independent given class. Although independence is generally a poor assumption, in practice naive Bayes often competes well with more sophisticated classifiers” ([80] [p. 41]). We chose this classifier in light of the view expressed in another study that “NB is the best choice under the condition of highly imbalanced class distribution” ([81] [p. 454]).
- RF trains various unpruned decision trees by iteratively sampling the original data set without replacement. Each tree is then used to classify an instance individually [38] and the instance is assigned to a class by counting. One of RF’s features is that it can calculate strength or importance measures using the *Out–of–Bag* (OOB) method, which enhances understanding of which attributes have greater predictive power. The only parameter that has to be chosen is *n*, the number of variables selected randomly in each node of the *N* available variables. The value of *n* is determined experimentally by selecting the value that minimizes the error rate for the OOB data. In our study the number of variables selected at random was *n* = 8 for WC and *n* = 3 for CN. For both data sets, RF was trained with 500 trees to grow to ensure every input row was predicted at least a few times. We chose this classifier because it performs well in databases with relatively few cases, high–dimensional feature space and complex data structures [82, 83].

#### Addressing unbalanced class distribution

A dataset is unbalanced if the classes are not more or less equally represented. This is true of both of our case studies. In WC, 7 of the 61 individuals in the network were found guilty while in CN, it was 14 out of 110. If the imbalance is not corrected it may result in very low levels on the recall and precision performance measures. To rebalance the two data sets we therefore applied the SMOTE approach [59], one of the most widely used strategies in the machine learning community for dealing with unbalanced classes in classification problems. This technique over–samples the minority class by creating synthetic examples rather than over–sampling with replacement. It is administered “by taking each minority class sample and introducing synthetic examples along the line segments joining any/all of the *k* minority class nearest neighbours. Depending upon the amount of over–sampling required, neighbours from the *k* nearest neighbours are randomly chosen” ([59] [p. 328]). SMOTE has been successfully used to balance classes in classification problems involving social network data [84–86]. Here, for the WC case SMOTE obtained 42 synthetic observations for class 1 and 70 for class 0 while in the CN case, the corresponding numbers were 84 and 140.

#### Model validation

In order to prevent overfitting, the models generated by LR, NB and RF were validated using the 10–fold–CV technique. Previous research has recommended the use of *k*–fold cross-validation sampling in networked data given that “the test accuracies of classifiers that use network information are always better. This is due to the fact that with random partition the nodes in the test partition will naturally have more neighbours from train and validation partitions, which have the actual labels, as opposed to the labels estimated by the classifiers” ([87] [p. 146]). The 10–CV technique has been used in cultural modelling, an emerging field aimed at developing computational models of small groups [81, 88–90]. The 10–CV randomly splits the original sample into 10 “folds” or subsamples. One of the nine subsamples is only used to *test* the model, while the remaining nine are used for the algorithm *training* process. This process is repeated 10 times for each of the *k* subsamples. Thus, 10 outcomes are obtained that are then averaged to evaluate the performance of the classifier.

### Performance measures

**Fig 4:**
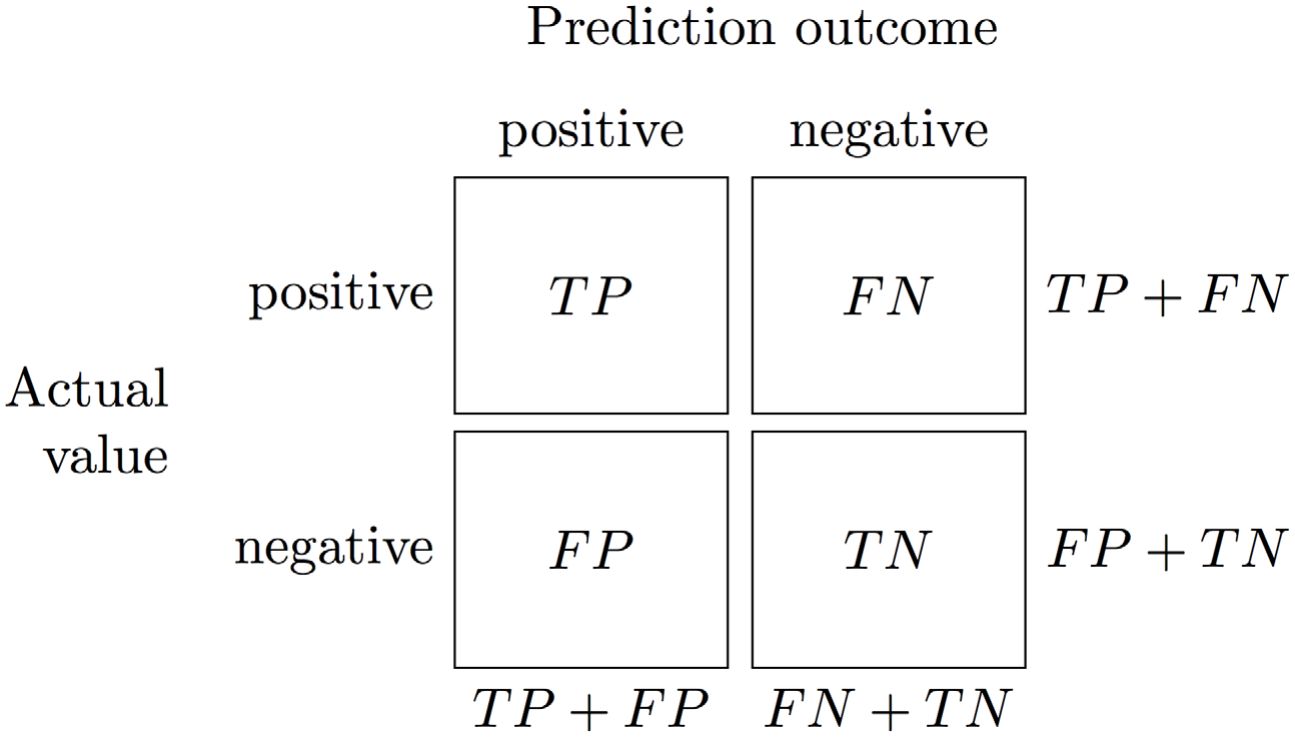
Confusion matrix for a two–class problem. *TP* is the number of correct predictions that an instance is positive (*true positive*), *FN* is the number of incorrect predictions that an instance is negative (*false negative*), *FP* is the number of incorrect predictions that an instance is positive (*false positive*) and *TN* is the number of correct predictions that an instance is negative (*true negative*).

The performance measures for the above techniques were calculated using a confusion matrix, that is, a matrix containing the numbers of positive and negative predictions made by a classification system (see Fig 4).

The three classifiers’ respective performances were evaluated using standard measures of *Accuracy*[61], *Precision*[62], *Recall*[62] and the Area under the ROC curve (*AUC*) [63, 64], the lattermost computed via *Leave–One–Out Cross–Validation* (LOOCV) [91] as suggested by Airola et al. [92, 93] for small data sets. Also applied was the *Matthews Correlation Coefficient* (MCC) [65], which is often used to measure performance with unbalanced databases (see Table 3).

**Table 3:** Performance measures for binary classification problems.

**Performance Measure**	**Formula**
**Accuracy**	$\frac{TP+TN}{TP+FP+FN+TN}$
**Precision**	$\frac{TP}{TP+FP}$
**Recall**	$\frac{TP}{TP+FN}$
**MCC**	$\frac{TP\cdot TN-FP\cdot FN}{\sqrt{\left(TP+FP)(TP+FN)(FP+TN)(FN+TN\right)}}$
**ROC Area**	$\frac{1}{2}\left(\frac{TP}{TP+FN}+\frac{TN}{TN+FP}\right)$

*Note*: Formulas are based on the confusion matrix classifications of Fig 4. Each measure varies between 0 and 1 except MCC, which takes values between −1 and +1. In every case, values close to +1 indicate a good performance.

### Statistical evaluation of models

Two tests were used to evaluate the performance of LR, NB and RF: Cochran’s *Q* test [94], which evaluates the three classifiers simultaneously, and McNemar’s test ($\chi_{Mc}^{2}$) [66], which evaluates them pair by pair. For Cochran’s *Q* the null hypothesis (*H*_0_) was that the three performed similarly whereas the alternative hypothesis (*H*_1_) was that they did not, that is, that they performed differently. For McNemar’s test, the null hypothesis (*H*_0_) was that the three models performed similarly while the alternative hypothesis (*H*_1_) was that their performances differed.

### Softwares

The social network measures were computed using the Organization Risk Analyzer (ORA) software tool [70, 95, 96]. We also used the following R packages for data analysis: DMwR R package for SMOTE [97], r-base-core for LR [98], e1071 R package for NB [99], RandomForest R package for RF and variable importance analysis [100], cvTools R package for computing 10–fold–CV and ROC with LOOCV [101], RVAideMemoire R package for the Cochran’s *Q* Test [102], and Package exact2x2 for McNemar’s test [103]. Questions or comments regarding the quantitative data analysis using the ORA and R Packages may be addressed to the authors.

## Results

In this section we present and compare the performance scores for the WC and CN cases, and also set out the importance values of the variables in the best predictive model obtained.

### Classification results for the WC case

**Fig 5:**
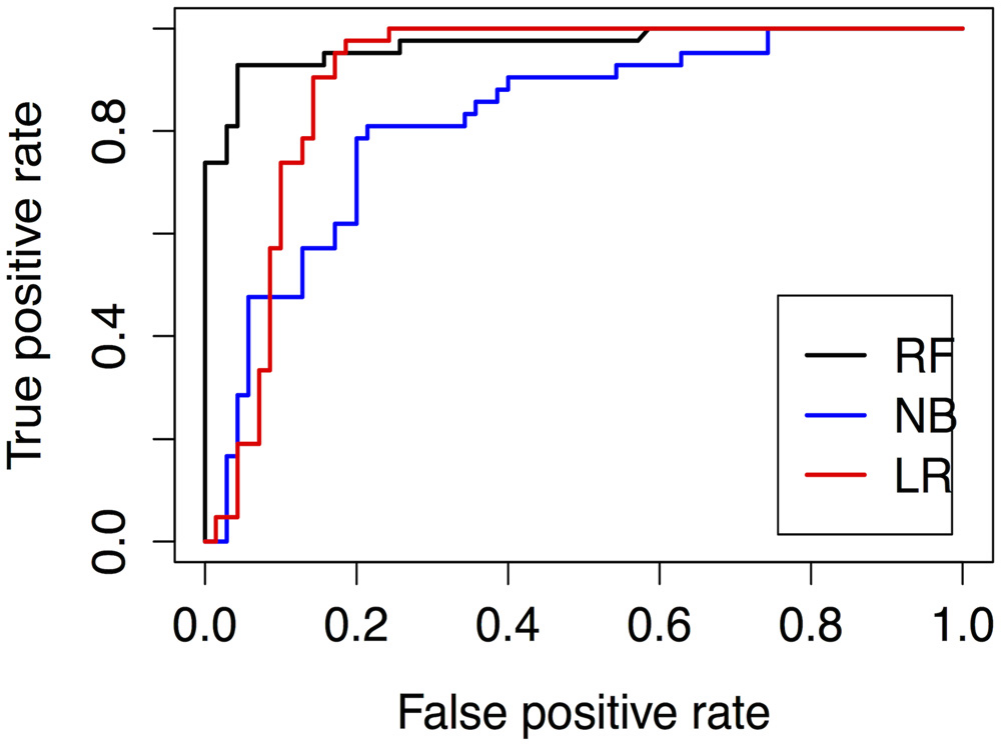
ROC curve for Watergate (using LOOCV).

The results for the WC case are summarized in Table 4, which shows the average performance scores (AVG) and their standard deviations (SD) for the three classification models. As can be seen, RF was the classifier with the highest average scores (in bold type) and the lowest standard deviations (underlined) for the accuracy, precision, recall, MCC measures, and ROC Area (AUC) (see Fig 5). Also apparent is that LR outperformed NB on all of the measures except Recall, where NB did better.

**Table 4:** Classifier performance in the WC case.

Performance measure	LR	NB	RF
Accuracy			
(AVG)	.866	.652	**.973**
(SD)	.077	.172	.044
Precision			
(AVG)	.794	.525	**.986**
(SD)	.182	.225	.045
Recall			
(AVG)	.860	.963	**.966**
(SD)	.198	.084	.074
MCC			
(AVG)	.880	.684	**.991**
(SD)	.079	.093	.003

Cochran’s *Q* test rejected the null hypothesis (*Q* = 5.09 with *p*<0.10), although only at the 10% significance level, meaning that performances of LR, NB and RF were statistically different. McNemar’s pair–by–pair test with continuity correction found that RF’s higher scores were significant in comparison to both NB (*H*_0_ is rejected, $\chi_{Mc}^{2}\left(1\right)$ = 29.6, *p* <.001) and LR (*H*_0_ is rejected, $\chi_{Mc}^{2}\left(1\right)$ = 20.3, *p* <.001). The tests also demonstrated that LR’s superior performance to NB was significant (*H*_0_ is rejected, $\chi_{Mc}^{2}\left(1\right)$ = 6.68, *p* = .0087). Clearly, then, RF was the classifier that performed best in modelling verdict outcomes in the WC case while LR did better than NB.

### Classification results for the CN case

**Fig 6:**
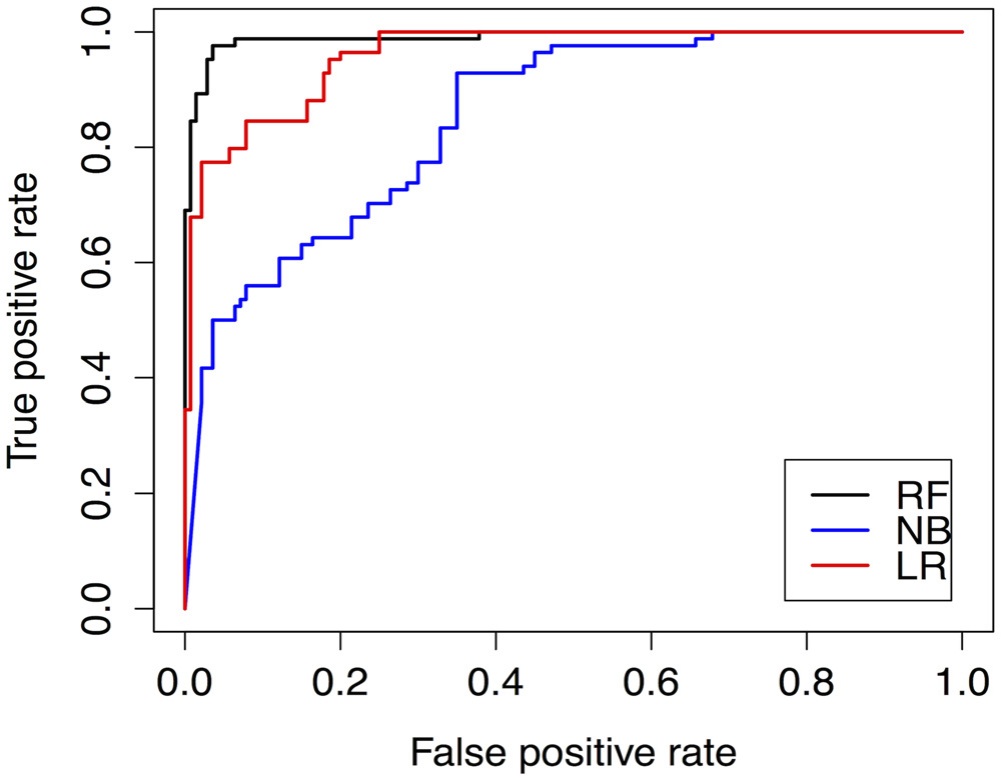
ROC curve for Caviar Network (using LOOCV).

The results in the CN case are summarized in Table 5. Once again, they show that RF was the classifier with the highest average performance scores (in bold) and the lowest standard deviations (underlined) for the accuracy, precision, recall, MCC measures, and ROC Area (AUC) (see Fig 6). LR outperformed NB on all of the measures except Precision, where NB did better.

**Table 5:** Classifier performance in the CN case.

Performance measure	LR	NB	RF
Accuracy			
(AVG)	.889	.785	**.951**
(SD)	.067	.067	.038
Precision			
(AVG)	.881	.904	**.943**
(SD)	.139	.130	.057
Recall			
(AVG)	.796	.507	**.979**
(SD)	.188	.143	.034
MCC			
(AVG)	.814	.734	**.999**
(SD)	.187	.147	.001

Cochran’s *Q* test rejected the null hypothesis (*Q* = 31.75 with *p*<0), meaning that performances of LR, NB and RF were statistically different. McNemar’s pair–by–pair test with continuity correction found that as in the WC case, RF’s higher performance scores were significant in comparison with both NB (*H*_0_ is rejected, $\chi_{Mc}^{2}\left(1\right)$ = 30.1, *p* < 0.001) and LR (*H*_0_ is rejected, $\chi_{Mc}^{2}\left(1\right)$ = 9.63, *p* < 0.001). The tests also showed that LR’s superior performance relative to NB was again significant (*H*_0_ is rejected, $\chi_{Mc}^{2}\left(1\right)$ = 6.68, *p* = .0087). Thus, in the CN case the RF classifier performed best in modelling verdict outcomes while LR did better than NB.

Since RF obtained the best results in both cases, the following subsection presents the importance values of the social network measures in the predictive models.

### Importance of social network measures in verdict classification

RF is used not only for classification but also to assess variable importance. The latter concept is defined as the total decrease in node impurities from splitting on the variable, averaged over all trees, where node impurity is measured by the Gini index [38, 104]. The variable with the highest index has the greatest impact on classifier performance of all the variables tested in correctly modelling what class each instance belongs to.

**Fig 7:**
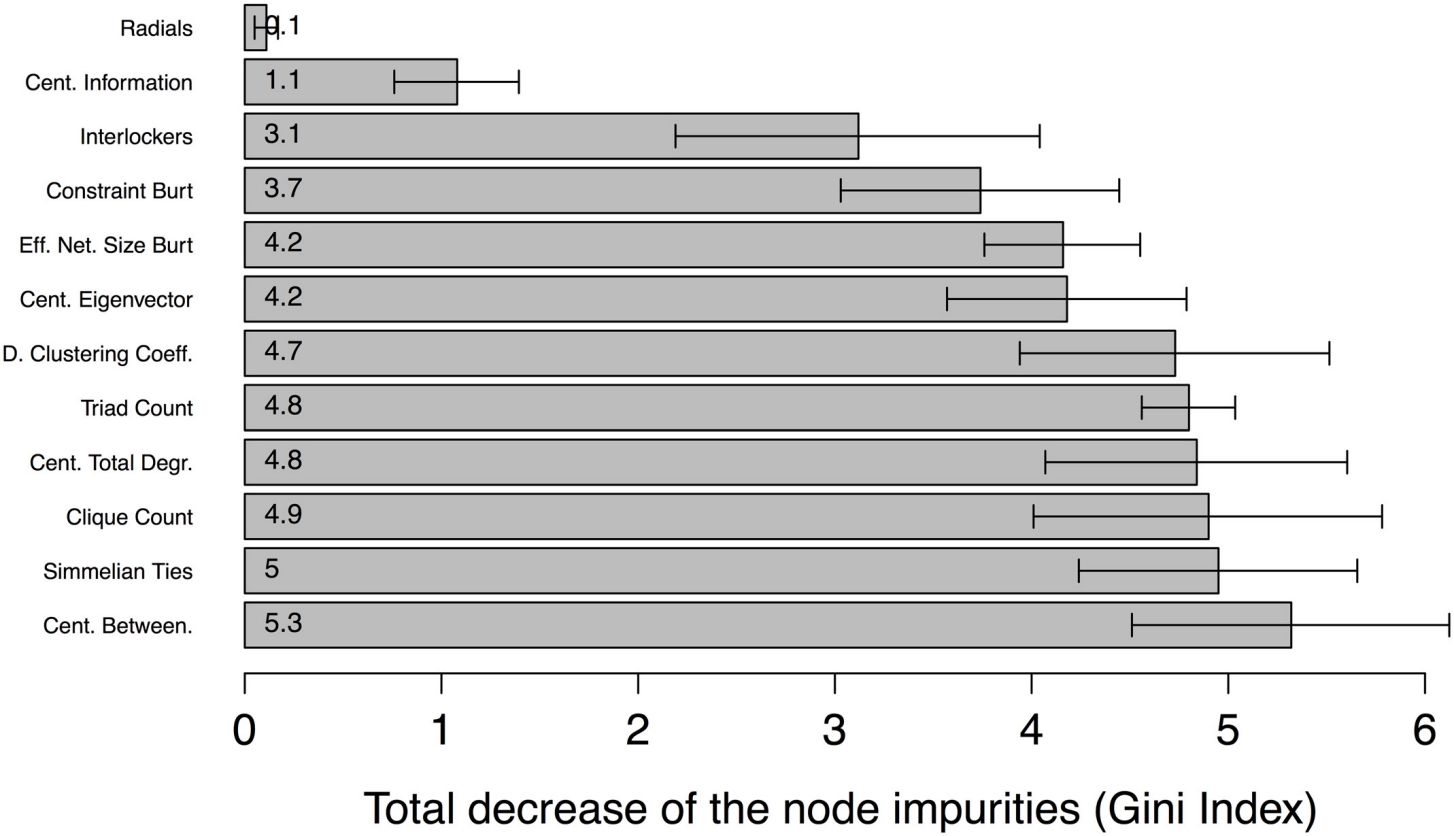
Variable importance by Gini index (means, lower and upper limits of 95% confidence interval) for modelling verdict outcome in WC case.

**Fig 8:**
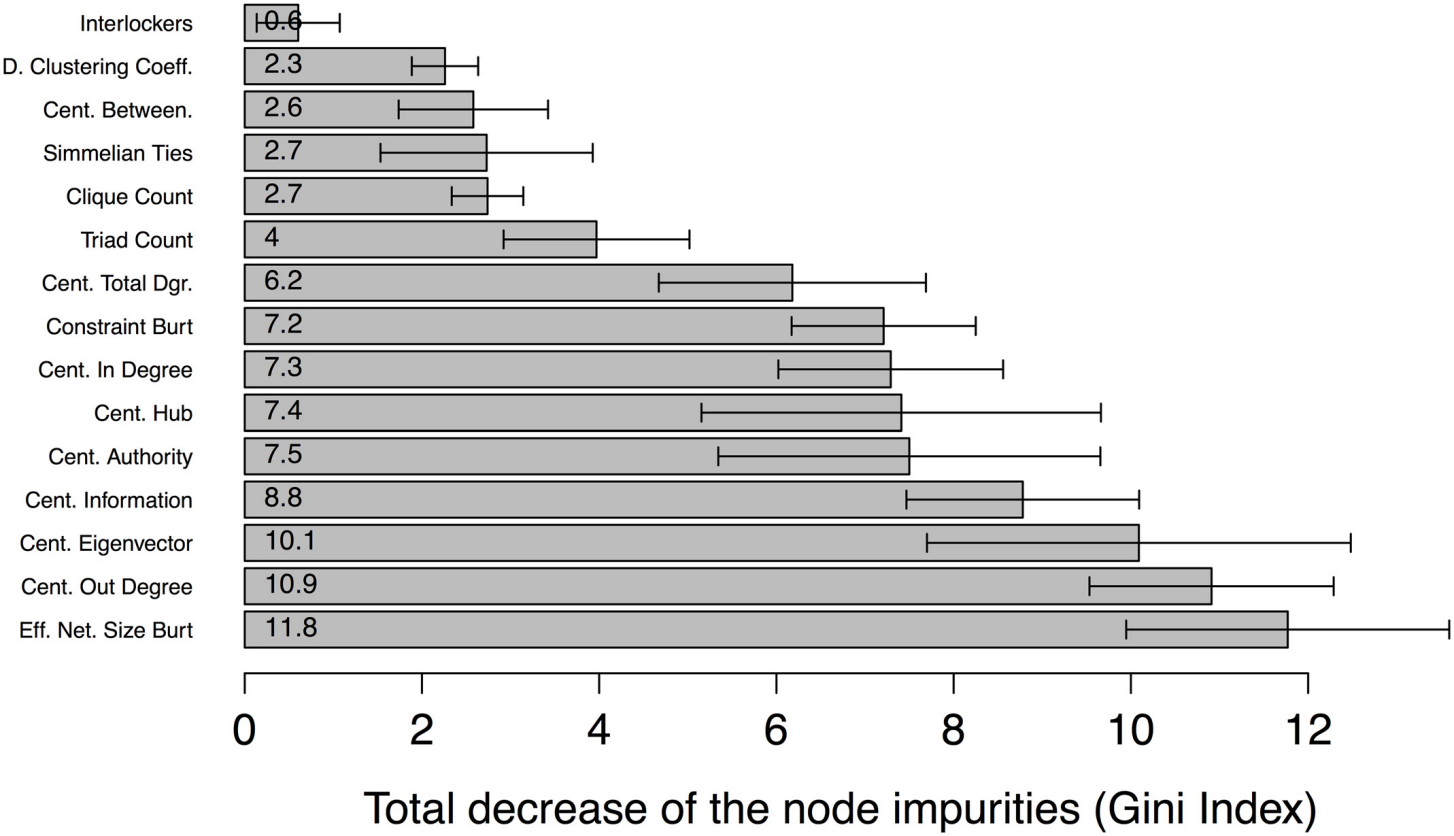
Variable importance by Gini index (means, lower and upper limits of 95% confidence interval) for modelling verdict outcome in CN case.

The importance analysis for RF was conducted following the procedure proposed by Breiman [38]. The permutation–based mean decrease in accuracy was used to measure the importance of each variable in the classification. The importance values for each variable in the WC and CN cases are displayed in Figs 7 and 8, respectively. In the WC case, centrality betweenness was the most important social network measure as measured by the Gini index in discriminating between innocent and guilty parties. The next five most important variables were the simmelian ties, clique count, degree centrality, triad count and clustering coefficient measures.

In the CN case, effective network size was the social network measure of greatest importance as indicated by the Gini index in discriminating between innocent and guilty parties. The next two in importance were out–degree centrality and then eigenvector centrality.

## Discussion

The results set out in the previous section demonstrated clearly that RF performed better, and in a statistically significant manner, than either NB or LR on the accuracy, recall, precision, MCC, and ROC measures. We may therefore conclude that RF produced better verdict outcome classifications in the two cases studied than the other two classifiers. But beyond this basic conclusion there are a number of important issues raised by the analysis presented thus far that are taken up in the following subsections.

### On social network measures

The three published works discussed here earlier [26–28] on modelling verdict outcomes with social network measures used only seven, one and four measures, respectively (see Table 1 above). This contrasts with the present study in which 16 were employed (see Table 2 above). If we compare the importance values we obtained for the measures using the RF model in the WC case with the original WC study [27], we find that they agree on the primary importance of betweenness centrality in determining verdict outcomes. The original study utilized this measure to operationalise the notion that “political conspiracies rely on brokers between individuals and mediators between groups to integrate the cabals with each other and cabals with the cadre” ([27] [p. 266]). We found, however, that betweenness centrality was not able to take directly into account the small groups in which actors play the role of broker or gatekeeper. Despite this measure’s importance, then, other measures pointing to the social microstructures that agents intermediate must also be investigated.

Our results showed that there are in fact several other measures with degrees of importance similar to that of betweenness centrality, such as simmelian ties, clique count, degree, triad count and clustering coefficient. All of these indicators attempt to measure network substructures, the very phenomenon [27] referred to in the observation quoted above on the WC case network being organized into *cabals* that are part of *cadres*. If we consider the WC study authors’ assertion that “[a] cabal is a clandestine team assembled to carry out political sabotage, espionage, and other illegal activities” ([27] [p. 266–267]), such social structures are precisely the sort that can be detected by the simmelian ties (ties embedded in cliques), clique count, degree, triad count and clustering coefficient measures. In the WC case network (i.e., a cadre), the agents initially found guilty were those who developed a high degree of betweenness and were organized into clandestine teams (i.e., a cabal).

If we compare the importance values of the RF model variables obtained above with the original investigation in the CN case [28], we find that while out–degree centrality was important in both studies, effective network size had greater importance in the RF model. This measure has been explored empirically in a paper on criminal networks in Québec, Canada by Morselli and Tremblay [105]. The two authors conducted a correlational analysis of the data gathered from a survey of inmate volunteers in southern Quebec prisons, finding that “higher proportions of nonredundancy in personal networks (networks with higher effective size) were positively associated with criminal earnings, market crime commissions and low self–control, and negatively related with the age of the offender”. And in a path analysis of the same data, Morselli, Tremblay and McCarthy corroborated that “mentorship increases effective size, which in turn increases criminal earnings. In other words, criminal mentors improve their protégés’ social capital and such brokerage–like networking offers a competitive advantage in crime” ([106] [p. 35]). Thus, both the present study and previous empirical evidence have found that the effective network size measure plays a prominent role in this type of criminal network.

The importance value obtained for the eigenvector centrality measure in the CN case also deserves comment. From a theoretical viewpoint, eigenvector centrality is designed to identify central agents connected to others who are also central. As one author has put it, “[e]igenvector centrality capitalizes on how differences in degree can propagate through a network (…) If one believes that differences in degree drive centrality, status, or power, then eigenvector centrality is called for” ([107] [p. 561]). Empirically, the measure has been successful in identifying criminals. For example, it was used as an index for ranking the “Men of Honor” among members of the U.S. Mafia and enabled the construction of a predictive model for detecting criminal leaders [108]. Eigenvector centrality has also been proposed for locating central agents in co–offending networks [109]. The measure’s importance in our RF model indicates the possibility of a hierarchical typology of criminal networks in which there are agents in high positions connected to many agents in low positions. Indeed, the three measures taken together suggest the following is true of CN: (a) the network has an organizational style in which the criminals control the effective size of their ego–network to distribute their earnings (effective network size); (b) it communicates directly with various agents (out–degree centrality); and (c) it has central agents that connect with other central agents (eigenvector centrality). In our view, the insights into the interplay between the various features of a social network are the most interesting aspect of importance analysis.

### Challenges Ahead

A criminal network can be studied using different analytical techniques. Current pedagogical practice, however, tends to give preference to conventional rather than alternative approaches. This is reflected in introductory textbooks on quantitative criminology, which present LR as one of the first techniques of choice in binary classification problems [110–112]. The present study provides evidence that at least one alternative method has the ability to generate predictive models which are highly competitive with those produced by LR in criminal responsibility identification. The results we obtained are consistent with a small group of studies that have also reported better performance for RF than for LR or NB [54, 113]. Further research is required to compare the performance of different classifiers in the data domain.

The number of social network measures with predictive potential that can be used to characterize a criminal network is constantly growing. The data domain of the social networks is based on two primitive types of data: (a) who a network member related with; and (b) how many times the relationship was activated. With these data types a virtually infinite number of social network indicators can be constructed. In the last three decades in particular, a number of new social network measures have been developed [44, 74, 114–120], including variations on the classic centrality measures [121–128]. In addition, *weighted* social networks measures have been created that enable human interaction to be explored in new ways (see [129] and [130]). However, the inclusion of weighted social network measures in prediction problems has the undesirable effect of increasing the dimensionality of the data. New machine learning approaches must therefore be developed or adapted for using this new type of measure. Other types of social network measures appear regularly in specialized journals such as *Social Networks* or *Connections*. New techniques for selecting and managing the growing dimensionality generated by this increasing variety of available measures will have to be developed in future research.

Difficulties may also arise with the use of classifiers for verdict classification if the database for the analysis has class imbalances [131], that is, if the dependent variable has a class with significantly more innocent than guilty parties or vice versa. In this study we used the SMOTE technique to balance the classes in both cases, but other strategies exist in the literature [88, 132–134]. Another potential complication has to do with the size of some criminal networks [109, 135]. The natural size of a human social network is ≈ 150 [136], or in more specific terms, “[m]aximum network size averaged 153.5 individuals, with a mean network size of 124.9 for those individuals explicitly contacted” ([137] [p. 53]). In criminal networks, however, with the exception of certain cases such as the Italian Mafia [108] or corrupt companies such as ENRON [138], the number of members may be relatively limited [139]. This means that future investigations will require techniques that can learn quickly from a small number of observations.

Finally, there is the question of which structural aspects of criminal networks influence the way social network measures differ in their importance in determining verdicts. In our results for the WC and CN cases, the order of importance of the social network measures is not the same. In qualitative terms, the variables which predicted verdict outcome were different. Whereas effective network size was eighth in importance in WC, it was first in CN. Centrality betweenness, meanwhile, was one of the least important in CN but the most important in WC. This is indirect evidence that the configuration of the two networks and their relationships with verdict outcome also varied. Further research such as that reported in [140] is needed to compare the structures of networks in terms of their topological characteristics in order to understand how structural aspects contribute to explaining criminal network verdict outcomes.

### Limitations of this experimental study

This experimental study has three main limitations. The first one has to do with the interpretation of the RF models. Despite their good classification performance, RF is a black box type of algorithm and the models are difficult for humans to interpret in the sense that the results do not indicate the individual effects of each attribute on the output variable. Future research should include an investigation of visualization techniques based on sensitivity analyses as suggested by [141].

The second limitation relates to the possible bias stemming from the treatment of unbalanced classes. Although SMOTE was used in the present study to address the class imbalance problem, the small number of available instances for training the algorithms may have produced an unknown level of bias in the synthetic classes generated. However, the results show that the models generated by RF exhibit low error levels in the classification results, a sign that SMOTE combined with RF helped to increase the predictive ability of the models in both classes. As noted early, several studies have applied SMOTE in classification problems using social network data. For example, it was used to create a model that predicts social security fraud detection in Belgium [86]. In another case the technique was utilized to reduce some of the effects of class imbalance among Trust and Distrust classes in social network online services [84]. Finally, it was employed to rebalance the classes for a trust prediction problem using social network data [85]. Additional research (for example, simulation studies using artificial generated data) to improve our understanding of the effect of SMOTE on class distribution would nevertheless be useful.

The third limitation of this study, a problem inherent in verdict classification, is that our models assumed the judicial system reached the correct verdict in each case. Yet it is well known that in the WC case, although Richard Nixon was one of the main perpetrators, the system treated him differently than the others. As for the CN case, some of the criminal agents were not convicted in exchange for informing on the others. Given the many complications in any criminal justice process, and more specifically, the complex rules governing criminal trials, the vagaries in the performance of the prosecution and defence teams and the quality of the evidence, both this and previous studies have had little choice but to proceed on the assumption that the criminal justice systems’ verdicts are correct. In other words, what is really being studied is not who was actually guilty but how the justice systems classify guilt. Thus, for determining guilt or innocence the methods we have discussed have real limitations, but for modelling the behaviour of the judicial process they have much to offer.

### Final words

This study has attempted to respond to a number of questions and define new tasks for modelling criminal trial verdicts using social networks measures. The ultimate goal is to provide criminologists with valuable feedback for the efficient allocation of resources and effort to issues of public interest. The application of machine learning in criminal networks requires further study, particularly as regards ethical and legal questions that arise in real–world cases. Greater application of social network analysis and machine learning in quantitative criminology could provide valuable information about the organization of criminal networks and their networked behaviour.

## Supporting Information

S1 Supporting Information Computed SNA measures. (ZIP)

## References

[1] Simester DI, Brodie RJ. Forecasting criminal sentencing decisions. International Journal of Forecasting. 1993;9(1):49–60. 10.1016/0169-2070(93)90053-P

[2] Morselli C. Crime and networks Criminology and Justice Studies. Taylor & Francis; 2013.

[3] Malm A, Bichler G. Networks of collaborating criminals: Assessing the structural vulnerability of drug markets. Journal of Research in Crime and Delinquency. 2011;48(2):271–297. 10.1177/0022427810391535.

[4] Calderoni F. The structure of drug trafficking mafias: the ‘Ndrangheta and cocaine. Crime, Law and Social Change. 2012;58(3):321–349. 10.1007/s10611-012-9387-9.

[5] Natarajan M. Understanding the structure of a large heroin distribution network: A quantitative analysis of qualitative data. Journal of Quantitative Criminology. 2006;22(2):171–192. 10.1007/s10940-006-9007-x

[6] Malm AE, Kinney JB, Pollard NR. Social network and distance correlates of criminal associates involved in illicit drug production. Security Journal. 2008;21(1):77–94. 10.1057/palgrave.sj.8350069

[7] Plecas D, Malm A, Kinney B. Marihuana growing operations in British Columbia revisited. Department of Criminology and Criminal Justice University College of the Fraser Valley 2005;p. 1–59.

[8] Klerks P. The network paradigm applied to criminal organizations: Theoretical nitpicking or a relevant doctrine for investigators? Recent developments in the Netherlands. Connections. 2001;24(3):53–65.

[9] Morselli C. Assessing vulnerable and strategic positions in a criminal network. Journal of Contemporary Criminal Justice. 2010;26(4):382–392. 10.1177/1043986210377105

[10] Sparrow MK. The application of network analysis to criminal intelligence: An assessment of the prospects. Social Networks. 1991;13(3):251–274. 10.1016/0378-8733(91)90008-H

[11] Faulkner RR, Cheney ER, Fisher GA, Baker WE. Crime by committee: Conspirators and company men in the Illegal Electrical Industry Cartel, 1954–19591. Criminology. 2003;41(2):511–554. 10.1111/j.1745-9125.2003.tb00996.x

[12] Davies T, Johnson S. Examining the relationship between road structure and burglary risk via quantitative network analysis. Journal of Quantitative Criminology. 2014;p. 1–27. Available from: 10.1007/s10940-014-9235-4.

[13] Masys A. Networks and network analysis for defence and security In: Proceedings of the 2013 IEEE/ACM International Conference on Advances in Social Networks Analysis and Mining. ACM; 2013 p. 1479–1480.

[14] Carley KM, Reminga J, Borgatti S. Destabilizing dynamic networks under conditions of uncertainty In: International Conference on Integration of Knowledge Intensive Multi–Agent Systems. Boston MA: IEEE KIMAS; 2003 p. 121–126.

[15] Krebs VE. Mapping networks of terrorist cells. Connections. 2002;24(3):43–52.

[16] Radil SM, Flint C, Tita GE. Spatializing social networks: Using social network analysis to investigate geographies of gang rivalry, territoriality, and violence in Los Angeles. Annals of the Association of American Geographers. 2010;100(2):307–326. 10.1080/00045600903550428

[17] Tita GE, Radil SM. Spatializing the social networks of gangs to explore patterns of violence. Journal of Quantitative Criminology. 2011;27(4):521–545. 10.1007/s10940-011-9136-8

[18] Young JT. How do they ‘end up together’? A social network analysis of self–control, homophily, and adolescent relationships. Journal of Quantitative Criminology. 2011;27(3):251–273. 10.1007/s10940-010-9105-7

[19] Haynie DL. Friendship networks and delinquency: The relative nature of peer delinquency. Journal of Quantitative Criminology. 2002;18(2):99–134. 10.1023/A:1015227414929

[20] Papachristos AV. The coming of a networked criminology. Advances in Criminological Theory. 2011;17:101–140.

[21] Young JT, Rees C. Social networks and delinquency in adolescence: Implications for life–course criminology In: Handbook of Life–Course Criminology. Springer; 2013 p. 159–180.

[22] Duijn PA, Klerks PP. Social network analysis applied to criminal networks: Recent developments in Dutch law enforcement In: Networks and Network Analysis for Defence and Security. Springer; 2014 p. 121–159.

[23] Calderoni F. Social network analysis of organized criminal groups In: Encyclopedia of Criminology and Criminal Justice. Springer Science + Business Media; 2014 p. 4972–4981. Available from: 10.1007/978-1-4614-5690-2_239

[24] Strang SJ. Network analysis in criminal intelligence In: Networks and Network Analysis for Defence and Security. Springer; 2014 p. 1–26.

[25] Hosseinkhani J, Koochakzaei M, Keikhaee S, Naniz JH. Detecting suspicion information on the Web using crime data mining techniques. International Journal of Advanced Computer Science and Information Technology. 2014;3(1):32–41.

[26] Baker WE, Faulkner RR. The social organization of conspiracy: Illegal networks in the Heavy Electrical Equipment Industry. American Sociological Review. 1993 12;58(6):837 10.2307/2095954

[27] Faulkner RR, Cheney ER. Breakdown of brokerage: Crisis and collapse in the Watergate conspiracy In: Morselli C, editor. Crime and Networks. Criminology and Justice Studies. Taylor & Francis; 2013 p. 263–284.

[28] Morselli C, Masías VH, Crespo F, Laengle S. Predicting sentencing outcomes with centrality measures. Security Informatics. 2013;2(1):1–9. 10.1186/2190-8532-2-4

[29] Masías VH, Valle MA, Amar JJ, Cervantes M, Brunal G, Crespo FA. Characterising the personality of the public Safety offender and non–offender using decision trees: The case of Colombia. Journal of Investigative Psychology and Offender Profiling. 2016;p. In press.

[30] Masías VH, Krause M, Valdés N, Pérez JC, Laengle S. Using decision trees to characterize verbal communication during change and stuck episodes in the therapeutic process. Frontiers in Psychology. 2015;6 10.3389/fpsyg.2015.00379 25914657 PMC4391223

[31] Japkowicz N, Shah M. Evaluating learning algorithms: A classification perspective. Cambridge University Press; 2011.

[32] Caruana R, Niculescu-Mizil A. An empirical comparison of supervised learning algorithms In: Proceedings of the 23rd international conference on Machine learning. ACM; 2006 p. 161–168.

[33] Fawcett T. An introduction to ROC analysis. Pattern Recognition Letters. 2006;27(8):861–874. 10.1016/j.patrec.2005.10.010

[34] Sun Y, Kamel MS, Wong AK, Wang Y. Cost–sensitive boosting for classification of imbalanced data. Pattern Recognition. 2007;40(12):3358–3378. 10.1016/j.patcog.2007.04.009

[35] Liu YY, Yang M, Ramsay M, Li XS, Coid JW. A comparison of logistic regression, classification and regression tree, and neural networks models in predicting violent re–offending. Journal of Quantitative Criminology. 2011;27(4):547–573. 10.1007/s10940-011-9137-7

[36] Hosmer DW Jr, Lemeshow S. Applied logistic regression. John Wiley & Sons; 2004.

[37] Langley P, et al An analysis of Bayesian classifiers In: Proceedings of the tenth national conference on Artificial intelligence. AAAI Press; 1992 p. 223–228.

[38] Breiman L. Random Forests. Machine Learning. 2001;45(1):5–32. 10.1023/A:1010933404324

[39] Koschtzki D, Lehmann KA, Peeters L, Richter S, Tenfelde-Podehl D, Zlotowski O. Centrality indices In: Brandes U, Erlebach T, editors. Network analysis. New York: Springer; 2005 p. 16–61.

[40] Kutler SI. Watergate: A brief history with documents. Wiley; 2010 Available from: http://books.google.cl/books?id=GLfOIBZNOF0C.

[41] Force USWSP. Watergate special prosecution force: Final report. Department of Justice, Watergate Special Prosecution Task Force; 1977. Available from: http://books.google.cl/books?id=Y_hFumC6ktcC.

[42] Force USWSP. Report: Watergate special prosecution force. U.S. Government Printing Office; 1975. Available from: http://books.google.cl/books?id=pLcVMgAACAAJ.

[43] Freeman LC. A set of measures of centrality based on betweenness. Sociometry. 1977;p. 35–41. 10.2307/3033543

[44] Wasserman S, Faust K. Social network analysis: Methods and applications. Cambridge: Cambridge University Press; 1994.

[45] Helms R. Modeling the politics of punishment: A conceptual and empirical analysis of “Law in Action” in criminal sentencing. Journal of Criminal Justice. 2009;37(1):10–20. 10.1016/j.jcrimjus.2008.12.004

[46] Spohn C, Holleran D. The Imprisonment penalty paid by young, unemployed black and hispanic male offenders. Criminology. 2000;38(1):281–306. 10.1111/j.1745-9125.2000.tb00891.x

[47] Spohn C, Gruhl J, Welch S. Effect of race on sentencing: A re–examination of an unsettled question. The Law & Society Review. 1981;16: 71–88. 10.2307/3053550

[48] Thomson RJ, Zingraff MT. Detecting sentencing disparity: Some problems and evidence. The American Journal of Sociology. 1981;86(4):869–880. 10.1086/227320

[49] Bushway SD, Piehl AM. The inextricable link between age and criminal history in sentencing. Crime & Delinquency. 2007;53(1):156–183. 10.1177/0011128706294444

[50] Myers SL Jr. Statistical tests of discrimination in punishment. Journal of Quantitative Criminology. 1985;1(2):191–218. Available from: 10.1007/bf01268626.

[51] Mitchell O. A meta–analysis of race and sentencing research: Explaining the inconsistencies. Journal of Quantitative Criminology. 2005;21(4):439–466. 10.1007/s10940-005-7362-7

[52] Skolnick P, Shaw JI. The OJ Simpson criminal trial verdict: Racism or status shield? Journal of Social Issues. 1997;53(3):503–516. Available from: 10.1111/j.1540-4560.1997.tb02125.x.

[53] Mabrey DJ. Tactical terrorism analysis: A comparative study of statistical learning techniques to predict culpability for terrorist bombings in two regional low–intensity conflicts. Ph.D Thesis. Sam Houston State University; 2006.

[54] Hill JB, Mabrey DJ, Miller JM. Modeling terrorism culpability: An event–based approach. The Journal of Defense Modeling and Simulation: Applications, Methodology, Technology. 2013;10(2):181–191. 10.1177/1548512912455470

[55] Hill J, Miller JM, Mabrey DJ. Classification of terrorist group events in the Philippines: Location, location, location. Journal of Policing, Intelligence and Counter Terrorism. 2010;5(2):41–54. 10.1080/18335300.2010.9686948

[56] Akyuz K, Armstrong T. Understanding the sociostructural correlates of terrorism in Turkey. International Criminal Justice Review. 2011;21(2):134–155. 10.1177/1057567711407332

[57] Ngo FT, Govindu R, Agarwal A. Assessing the predictive utility of Logistic Regression, Classification and Regression Tree, Chi–Squared Automatic Interaction Detection, and Neural Network Models in predicting inmate misconduct. American Journal of Criminal Justice. 2014;p. 1–28. Available from: 10.1007/s12103-014-9246-6.

[58] Graham S, Ruths D, Bronk C, Subramanian D. The event–participant inference problem: Using open source information and Bayes’ rule to select for the most likely participants in a terrorist incident. White paper; 2009.

[59] Chawla NV, Bowyer KW, Hall LO, Kegelmeyer WP. SMOTE: Synthetic Minority Over–sampling Technique. Journal of Artificial Intelligence Research. 2002;16:321–357.

[60] Purushotham S, Tripathy BK. Evaluation of classifier models using stratified tenfold Cross Validation techniques In: Krishna PV, Babu MR, Ariwa E, editors. Global Trends in Information Systems and Software Applications. vol. 270 of Communications in Computer and Information Science. Springer Berlin Heidelberg; 2012 p. 680–690. Available from: 10.1007/978-3-642-29216-3_74.

[61] Sammut C, Webb GI. Accuracy In: Encyclopedia of Machine Learning. Springer US; 2010 p. 9–10.

[62] Ting K. Precision and Recall In: Sammut C, Webb G, editors. Encyclopedia of Machine Learning. Springer US; 2010 p. 781–781.

[63] Sammut C, Webb G. Area Under Curve In: Encyclopedia of Machine Learning. Springer US; 2010 p. 40–40.

[64] Bradley AP. The use of the area under the ROC curve in the evaluation of machine learning algorithms. Pattern Recognition. 1997;30(7):1145–1159. 10.1016/S0031-3203(96)00142-2

[65] Matthews BW. Comparison of the predicted and observed secondary structure of T4 phage lysozyme. Biochimica et Biophysica Acta (BBA)–Protein Structure. 1975;405(2):442–451. 10.1016/0005-2795(75)90109-9 1180967

[66] Dietterich TG. Statistical tests for comparing supervised classification learning algorithms. Oregon State University Technical Report. 1996;1:1–24.

[67] Bostanci B, Bostanci E. An evaluation of classification algorithms using Mc Nemar’s Test In: Proceedings of Seventh International Conference on Bio–Inspired Computing: Theories and Applications (BIC–TA 2012) Springer; 2013 p. 15–26.

[68] Faulkner RR, Cheney ER. The multiplexity of political conspiracy: Illegal networks and the collapse of Watergate. Global Crime. 2013;14(2–3):197–215. 10.1080/17440572.2013.790313

[69] Morselli C. Inside criminal networks. Springer; 2008.

[70] Carley KM, Pfeffer J, Reminga J, Storrick J, Columbus D. ORA User’s Guide 2013. DTIC Document; 2013.

[71] Bonacich P. Power and centrality: A family of measures. The American Journal of Sociology. 1987;92(1):11170–1182 Available from: 10.1086/228631.

[72] Kleinberg JM. Authoritative sources in a hyperlinked environment. Journal of the Association for Computing Machinery. 1999;46(5):604–632. 10.1145/324133.324140

[73] Stephenson K, Zelen M. Rethinking centrality: Methods and examples. Social Networks. 1989;11(1):1–37. 10.1016/0378-8733(89)90016-6

[74] Carley K. Summary of key network measures for characterizing organizational architectures. Unpublished Document: CMU. 2002;.

[75] Bron C, Kerbosch J. Algorithm 457: Finding all cliques of an undirected graph. Communications of the ACM. 1973;16(9):575–577. 10.1145/362342.362367

[76] Burt RS. Structural holes: The social structure of competition. Cambridge: Harvard University Press; 1995.

[77] Watts DJ, Strogatz SH. Collective dynamics of ‘small–world’ networks. Nature. 1998;393(6684):440–442. 10.1038/30918 9623998

[78] Krackhardt D. Simmelian ties: Super strong and sticky In: Power and Influence in Organizations. Thousand Oaks, CA: Sage;. p. 21–38. Available from: 10.4135/9781483345291.n2.

[79] Lewis DD. Naive (Bayes) at forty: The independence assumption in information retrieval In: Machine learning: ECML–98. Springer; 1998 p. 4–15.

[80] Rish I. An empirical study of the naive Bayes classifier In: IJCAI 2001 workshop on empirical methods in artificial intelligence. vol. 3 IBM New York; 2001 p. 41–46.

[81] Su P, Mao W, Zeng D. An empirical study of cost–sensitive learning in cultural modeling. Information Systems and e–Business Management. 2013;11(3):437–455. 10.1007/s10257-012-0198-4

[82] Qi Y. Random forest for bioinformatics In: Ensemble machine learning. Springer; 2012 p. 307–323.

[83] Caruana R, Karampatziakis N, Yessenalina A. An empirical evaluation of supervised learning in high dimensions In: Proceedings of the 25th international conference on Machine learning. ACM; 2008 p. 96–103.

[84] Graña M, Nuñez-Gonzalez JD, Ozaeta L, Kamińska-Chuchmała A. Experiments of trust prediction in social networks by artificial neural networks. Cybernetics and Systems. 2015;46(1–2):19–34. Available from: 10.1080/01969722.2015.1007725.

[85] Nuñez-Gonzalez JD, Graña M, Apolloni B. Reputation features for trust prediction in social networks. Neurocomputing. 2015;p. 1–7. 10.1016/j.neucom.2014.10.099

[86] Van Vlasselaer V, Meskens J, Van Dromme D, Baesens B. Using social network knowledge for detecting spider constructions in social security fraud In: Advances in Social Networks Analysis and Mining (ASONAM), 2013 IEEE/ACM International Conference on. IEEE; 2013 p. 813–820.

[87] Çataltepe Z, Sönmez A. Classification in social networks In: Social Networks: Analysis and Case Studies. Springer; 2014 p. 127–148.

[88] Su P, Mao W, Zeng D, Li X, Wang FY. Handling class imbalance problem in cultural modeling In: Intelligence and Security Informatics, 2009. ISI’09. IEEE International Conference on. IEEE; 2009 p. 251–256.

[89] Li XC, Mao WJ, Zeng D, Su P, Wang FY. Performance evaluation of machine learning methods in cultural modeling. Journal of Computer Science and Technology. 2009;24(6):1010–1017. 10.1007/s11390-009-9290-8

[90] Li X, Mao W, Zeng D, Su P, Wang FY. Performance evaluation of classification methods in cultural modeling In: Intelligence and Security Informatics, 2009. ISI’09. IEEE International Conference on. IEEE; 2009 p. 248–250.

[91] Sammut C, Webb G. Leave–One–Out Cross–Validation In: Sammut C, Webb G, editors. Encyclopedia of Machine Learning. Springer US; 2010 p. 600–601. Available from: 10.1007/978-0-387-30164-8_469.

[92] Airola A, Pahikkala T, Waegeman W, De Baets B, Salakoski T. An experimental comparison of cross–validation techniques for estimating the area under the ROC curve. Computational Statistics & Data Analysis. 2011;55(4):1828–1844. 10.1016/j.csda.2010.11.018

[93] Airola A, Pahikkala T, Waegeman W, De Baets B, Salakoski T. A comparison of AUC estimators in small–sample studies. In: 3rd International workshop on Machine Learning in Systems Biology (MLSB 09); 2009. p. 15–23.

[94] Sheskin DJ. Handbook of parametric and nonparametric statistical procedures. CRC Press; 1997.

[95] Wei W, Pfeffer J, Reminga J, Carley KM. Handling weighted, asymmetric, self–looped, and disconnected networks in ORA. DTIC Document; 2011.

[96] Qiuju Y, Qingqing C. A social network analysis platform for Organizational Risk Analysis–ORA In: Intelligent System Design and Engineering Application (ISDEA), 2012 Second International Conference on. IEEE; 2012 p. 760–763.

[97] Torgo L, Torgo ML. Package ‘DMwR’. 2013;Available from: https://cran.r-project.org/web/packages/DMwR/index.html.

[98] Ledolter J. Data mining and business analytics with R. John Wiley & Sons; 2013.

[99] Dimitriadou E, Hornik K, Leisch F, Meyer D, Weingessel A, Leisch MF. The e1071 package. Misc Functions of Department of Statistics (e1071), TU Wien. 2006;Available from: https://cran.r-project.org/web/packages/e1071/index.html.

[100] Liaw A, Wiener M. Classification and regression by Random Forest. R news. 2002;2(3):18–22.

[101] Alfons A. A toolkit for cross–validation: The R package cvTools. useR! The 8th International R User Conference, June 12–15, 2012, Nashville, Tennessee, USA. 2012;.

[102] Hervé M. RVAideMemoire: diverse basic statistical and graphical functions. R package version 09–32. 2014;Available from:https://cran.r-project.org/web/packages/RVAideMemoire/index.html.

[103] Fay MP. exact2x2: Exact conditional tests and matching confidence intervals for 2 by 2 tables. 2015;Available from: https://cran.r-project.org/web/packages/exact2x2/index.html.

[104] Archer KJ, Kimes RV. Empirical characterization of random forest variable importance measures. Computational Statistics & Data Analysis. 2008;52(4):2249–2260. 10.1016/j.csda.2007.08.015

[105] Morselli C, Tremblay P. Criminal achievement, offender networks and the benefits of low self–control. Criminology. 2004;42(3):773–804. 10.1111/j.1745-9125.2004.tb00536.x

[106] Morselli C, Tremblay P, McCarthy B. Mentors and criminal achievement. Criminology. 2006;44(1):17–43. 10.1111/j.1745-9125.2006.00041.x

[107] Bonacich P. Some unique properties of eigenvector centrality. Social Networks. 2007;29(4):555–564. 10.1016/j.socnet.2007.04.002

[108] Mastrobuoni G, Patacchini E. Organized crime networks: An application of network analysis techniques to the American mafia. Review of Network Economics. 2012;11(3):1–43. 10.1515/1446-9022.1324

[109] Tayebi MA, Bakker L, Glasser U, Dabbaghian V. Locating central actors in co–offending networks In: Advances in Social Networks Analysis and Mining (ASONAM), 2011 International Conference on. IEEE; 2011 p. 171–179.

[110] Piquero AR, Weisburd D. Handbook of quantitative criminology. Springer; 2010.

[111] Dantzker ML, Hunter RD. Research methods for criminology and criminal justice. Jones & Bartlett Learning; 2011.

[112] Walker JT, Maddan S. Statistics in criminology and criminal justice. Jones & Bartlett Learning; 2012.

[113] Berk R. Criminal justice forecasts of risk: A machine learning approach. New York: Springer; 2012.

[114] Newman MEJ. Mathematics of networks In: Networks. Oxford University Press (OUP); 2010 p. 109–167. Available from: 10.1093/acprof:oso/9780199206650.003.0006.

[115] Aggarwal CC. Social network data analytics. 1st ed Aggarwal CC, editor. Springer Science + Business Media; 2011.

[116] Brandes U, Erlebach T. Network analysis: Methodological foundations. vol. 3418 Springer; 2005.

[117] Costa LdF, Rodrigues FA, Travieso G, Villas Boas P. Characterization of complex networks: A survey of measurements. Advances in Physics. 2007;56(1):167–242. 10.1080/00018730601170527

[118] Xu K, Tang C, Tang R, Ali G, Zhu J. A comparative study of six software packages for complex network research In: Communication Software and Networks, 2010. ICCSN’10. Second International Conference on. IEEE; 2010 p. 350–354.

[119] Gibbons A. Algorithmic graph theory. Cambridge University Press; 1985.

[120] White S, Smyth P. Algorithms for estimating relative importance in networks In: Proceedings of the ninth ACM SIGKDD international conference on Knowledge discovery and data mining. ACM; 2003 p. 266–275.

[121] Brandes U. On variants of shortest–path betweenness centrality and their generic computation. Social Networks. 2008;30(2):136–145. 10.1016/j.socnet.2007.11.001

[122] Freeman LC, Borgatti SP, White DR. Centrality in valued graphs: A measure of betweenness based on network flow. Social Networks. 1991;13(2):141–154. 10.1016/0378-8733(91)90017-N

[123] Szczepański PL, Michalak T, Rahwan T. A new approach to betweenness centrality based on the shapley value. In: Proceedings of the 11th International Conference on Autonomous Agents and Multiagent Systems–Volume 1. International Foundation for Autonomous Agents and Multiagent Systems; 2012. p. 239–246.

[124] Pfeffer J, Carley KM. k–Centralities: local approximations of global measures based on shortest paths In: Proceedings of the 21st international conference companion on World Wide Web. ACM; 2012 p. 1043–1050.

[125] Borgatti SP. Centrality and network flow. Social Networks. 2005;27(1):55–71. 10.1016/j.socnet.2004.11.008

[126] Brandes U. A faster algorithm for betweenness centrality. Journal of Mathematical Sociology. 2001;25(2):163–177. 10.1080/0022250X.2001.9990249

[127] Newman ME. A measure of betweenness centrality based on random walks. Social Networks. 2005;27(1):39–54. 10.1016/j.socnet.2004.11.009

[128] De Meo P, Ferrara E, Fiumara G, Ricciardello A. A novel measure of edge centrality in social networks. Knowledge-Based Systems. 2012;30:136–150. 10.1016/j.knosys.2012.01.007

[129] Ballester C, Calvó-Armengol A, Zenou Y. Who’s who in networks. Wanted: The key player. Econometrica. 2006;74(5):1403–1417. 10.1111/j.1468-0262.2006.00709.x

[130] Opsahl T, Agneessens F, Skvoretz J. Node centrality in weighted networks: Generalizing degree and shortest paths. Social Networks. 2010;32(3):245–251. 10.1016/j.socnet.2010.03.006

[131] Berk R. Asymmetric loss functions for forecasting in criminal justice settings. Journal of Quantitative Criminology. 2011;27(1):107–123. 10.1007/s10940-010-9098-2

[132] Su P, Mao W, Zeng D. An empirical study of cost–sensitive learning in cultural modeling. Information Systems and e–Business Management. 2013;11(3):437–455. 10.1007/s10257-012-0198-4

[133] Berk R. Balancing the costs of forecasting errors in parole decisions. Albany Law Review. 2010;74:1071–1086.

[134] Kotsiantis S, Kanellopoulos D, Pintelas P. Handling imbalanced datasets: A review. GESTS International Transactions on Computer Science and Engineering. 2006;30(1):25–36.

[135] Sparrow MK. The application of network analysis to criminal intelligence: An assessment of the prospects. Social Networks. 1991;13(3):251–274. 10.1016/0378-8733(91)90008-H

[136] Dunbar RI. Coevolution of neocortical size, group size and language in humans. Behavioral and Brain Sciences. 1993;16(4):681–693. 10.1017/S0140525X00032325

[137] Hill RA, Dunbar RI. Social network size in humans. Human Nature. 2003;14(1):53–72. 10.1007/s12110-003-1016-y 26189988

[138] Klimt B, Yang Y. The Enron corpus: A new dataset for email classification research In: Machine learning: ECML 2004. Springer; 2004 p. 217–226.

[139] Bouchard M, Ouellet F. Is small beautiful? The link between risks and size in illegal drug markets. Global Crime. 2011;12(1):70–86. 10.1080/17440572.2011.548956

[140] Airoldi EM, Bai X, Carley KM. Network sampling and classification: An investigation of network model representations. Decision Support Systems. 2011;51(3):506–518. 10.1016/j.dss.2011.02.014 21666773 PMC3110739

[141] Cortez P, Embrechts MJ. Opening black box data mining models using sensitivity analysis In: 2011 IEEE Symposium on Computational Intelligence and Data Mining (CIDM). Institute of Electrical & Electronics Engineers (IEEE); 2011 p. 341–348. Available from: 10.1109/cidm.2011.5949423.

